# Exploring the Psychological Impact of Quarantine: An Investigation into Eating Patterns, Emotions, and Loneliness During the COVID-19 Pandemic in Greece

**DOI:** 10.7759/cureus.58411

**Published:** 2024-04-16

**Authors:** Nikolia Savvopoulou, Konstantinos Asimakopoulos, Philippos Gourzis, Eleni Jelastopulu

**Affiliations:** 1 Department of Public Health, School of Medicine, University of Patras, Patras, GRC; 2 Department of Psychiatry, School of Medicine, University of Patras, Patras, GRC

**Keywords:** loneliness, negative emotions, positive emotions, eating habits, quarantine

## Abstract

Purpose: The implementation of quarantine measures in response to the COVID-19 pandemic presented challenges linked to adverse psychological effects, notably affecting individuals' eating patterns. This study aimed to investigate the eating patterns of individuals during lockdowns compared across sex, age, and income levels, and examine the influence of positive and negative emotions, as well as loneliness, on these patterns.

Methods: A cross-sectional online study was conducted with 450 participants (aged 18-74 years old). One questionnaire about demographics, the Demographic Questionnaire, and three validated self-report scales (Eating Attitudes Test, comprising the Dieting, Bulimia, and Food Preoccupation, and Oral Control subscales, the Modified Differential Emotions Scale, and the UCLA Loneliness Scale) were employed. Convenience and snowball sampling were used. The data were collected between April and May 2021, primarily through social media platforms, such as Facebook, Instagram, and Twitter. The survey questionnaire was shared via these platforms and users could answer if they wanted. Also, they were asked to send the questionnaire to their close contacts. Additionally, the survey questionnaire was distributed face-to-face to 80 participants. The statistical analyses included linear regression and mediation analyses.

Results: Abnormal eating patterns *(*e.g. eating behaviors that tend to have signs of diet such as constant avoidance of fattening foods, the individual's involvement with becoming leaner, extreme control or preoccupation with food, overeating and purging methods*)* were identified in 25% of the 450 participants participated in this survey, aged 18-74 years. Moderate levels of negative/positive emotions and loneliness were predominantly reported. Female sex was significantly associated with abnormal eating patterns (p=0.010), particularly dietary behaviors (p=0.029). Negative emotions (p=0.032) and loneliness (p=0.001) emerged as predictive factors for overall eating patterns and bulimic behaviors. Negative emotions exhibited a direct correlation with eating patterns, while loneliness played a significant mediating role (p=0.032). Furthermore, the association between negative emotions and bulimia was partially mediated by loneliness (p=0.018).

Conclusions: This study underscores the pivotal roles of negative emotions and loneliness in shaping eating patterns during quarantine. Multilevel public health interventions are needed to address the negative effects of quarantine and pandemics in general. Screening tests for mental health in the school and job environments could highlight the need for shaping interventions, such as counseling, group empowerment, and family support in order to mitigate the negative impact of the COVID-19 pandemic on eating behaviors and mental health in general.

## Introduction

The COVID-19 pandemic has been recognized as one of the most significant public health events in recent years. Investigating the effects of the pandemic and its long-term consequences, in conjunction with the medical implications, has become a priority for the research community.

The COVID-19 pandemic affected not only the physical health but also the mental health and well-being of individuals [1]. As quarantine became the new reality for many people, it appeared to trigger symptoms of anxiety and stress, according to several related studies [2-5]. Women were found to have more frequently occurring symptoms of stress, anxiety, and depression than men [3, 6]. Furthermore, it has been found that female sex is strongly associated with loneliness [7] and insomnia [2] and is also generally associated with lower levels of mental health [8, 9].

Quarantine can be classified as a stressful event, and in general, such events tend to affect the eating patterns of individuals [10]. There seems to be a clear link between eating behaviors and emotional factors. Stress leads to overeating, especially sugar-rich "leisure foods", such as sweets and snacks [11]. These foods, especially those rich in simple carbohydrates, reduce stress as they encourage the production of serotonin, which has a positive effect on mood [12]. It is well known that experiencing negative emotions can lead to overeating, referred to as “emotional eating”. On the other hand, negative experiences can lead to food restriction due to the stress response that usually causes decreased appetite. During quarantine, there was an increase in food intake, while food overconsumption was used as a coping strategy to address grueling situations during the pandemic [13-16].

Increased social isolation and loneliness need to be included among the most significant consequences of the COVID-19 pandemic [17]. The risk factors associated with a greater sense of loneliness are young age, low income, unemployment, a lower level of education, smaller family size, living alone, reduced social support, physical or mental difficulty, difficulties regulating emotions, insomnia, and depression and anxiety [7, 18, 19]. The link between loneliness and eating disorders extends across the spectrum of disorders, from anorexia nervosa to episodic overeating and obesity, because loneliness plays an important role in food reduction and food addiction [20, 21].

Many studies revealed changes in eating patterns that were related to stress and negative emotions during the quarantine period [16,22]. Nonetheless, exploring the role of both negative and positive emotions, which is less studied, requires further research. Moreover, the effect of loneliness, a major condition, especially during quarantine, seems to be less studied in relation to the eating patterns of individuals. Our aim was to investigate the eating patterns of individuals during quarantine and the role of positive and negative emotions as well as loneliness in shaping these patterns. Abnormal eating patterns are defined patterns that include eating behaviors that tend to have signs of dieting such as constant avoidance of fattening foods as well as the individual's involvement with becoming leaner, extreme control or preoccupation with food, overeating, and purging methods. Abnormal eating patterns may indicate the existence of an eating disorder, but this needs further mental assessment. Contradictorily, normal eating patterns include behaviors where food consumption is based on one's nutritional needs without restrictions, preoccupations, or obsessions with food. Our main research hypotheses were as follows:

Firstly, sex, age, and income would influence the eating patterns of individuals. Secondly, emotions and loneliness would be predictive factors of eating patterns. Thirdly, loneliness would mediate the relationship between emotions and eating patterns.

## Materials and methods

Survey method

An online cross-sectional study was performed and included 450 individuals (n= 450, aged 18-74 years). Baseline data were collected between April and May 2021, mainly from social media platforms using self-report scales about demographics, eating attitudes, emotions, and loneliness. In addition, the survey questionnaire was distributed face to face to 80 participants in order to increase the sample size, as the responses from the online media did not show a steady rate. The sampling methods used were mixed: firstly, convenience sampling was used because the survey questionnaire was distributed through online groups on Facebook with lots of members where authors had free access, such as groups of students at Greek Universities, groups related to job and education issues, and art groups as well as on other social media platforms, specifically, Instagram and Twitter, primarily used by adults. Secondly, a snowball sampling procedure was also used, as participants were invited to send the questionnaire to their close contacts. Additional convenience sampling was employed as the survey questionnaire that was distributed face to face was also sent to close contacts of the authors. Out of 90 participants, only 10 declined to participate in the face-to-face session. Regarding the survey questionnaire that was shared via social media platforms, we have no information regarding how many persons had seen the invitation and avoided participating, as the invitation was free. The eligibility criterion was an age of at least 18 years. No other exclusion criteria were set, so the possibility of some of the participants having a psychiatric diagnosis had not been taken into consideration and this could have affected the outcome. Participants provided their consent online or face to face before completing the survey questionnaire in the Greek language, and no compensation was offered.

Measures

Demographic Questionnaire

For the purpose of the study, we used the survey questionnaire which consists of four self-reported scales in order to collect the data that were needed. Firstly, an initial questionnaire about demographics was used to collect basic information such as age, sex, and socioeconomic status.

Eating Attitudes Test

The Eating Attitudes Test (EAT-26) developed by Garner and Garfinkel was used to assess eating habits [23]. We used the Greek version of the test [24]. The instrument is a self-report questionnaire consisting of 26 items on a six-point Likert-type scale. The EAT-26 consists of three different subscales: the first subscale is the Diet subscale (13 questions), which assesses the degree of avoidance of fattening foods and the individual's involvement with becoming leaner; the second subscale is the Bulimia and Food Intake subscale (six questions), which evaluates the presence of repetitive and uncontrolled thoughts about food as well as the symptoms of bulimia such as overeating or purging methods; and the third subscale is the Oral Control subscale (seven questions), which assesses the degree of self-control around food intake as well as the perception of pressure from others to gain weight. The Diet subscale (13 questions) includes items regarding intense thinking about having a lean body and avoiding fatty foods; the Bulimia subscale (six questions) includes items regarding a lack of self-control in food intake and the occurrence of bulimic symptoms; and (the Oral control subscale (7 questions). The cut-off score is 20, and a score > 20 indicates abnormal eating habits. Regarding the psychometric properties of the questionnaire, the reliability of the scale is considered high, as the Cronbach’s alpha (α) index is 0.81 for the overall scale, 0.81 for the diet subscale, 0.62 for the bulimia subscale and 0.58 for the oral control subscale. In our study, the Cronbach’s alpha index for the overall scale was 0.751, that for the Diet subscale was 0.778, that for the Bulimia subscale was 0.697, and that for the Oral Control subscale was 0.621.

Modified Differential Emotions Scale

The Modified Differential Emotions Scale (DES.MOD, Fredrickson et al., 2003 [25]) asks participants to recall the past two weeks and rate their strongest experience of 20 specific emotions on a 5-point Likert scale (1-Not At All to 5-Extremely). We used the Greek version of the instrument [26]. The Positive Emotions subscale is a composite of nine positive emotions, and the Negative Emotions subscale is a composite of seven negative emotions (all but embarrassment), with a coefficient of α = 0.69. Cut-off scores have not been defined. In this study, the Cronbach’s alpha index for the Positive Emotion subscale was 0.896, and that for the Negative Emotions subscale was 0.824.

Revised UCLA Loneliness Scale

The Revised UCLA Loneliness Scale is used to measure an individual’s subjective feelings of loneliness as well as feelings of social isolation [27]. The instrument is a self-report questionnaire consisting of 20 items scored on a four-point Likert-type scale. The scale has been adapted to a Greek sample [28]. Cut-off scores have not been defined. Regarding the psychometric properties of the questionnaire, the reliability is considered high, as the Cronbach’s alpha (α) index is 0.89. In this study, the Cronbach’s alpha index was 0.922.

Statistical analyses

Descriptive statistics were employed to explore the demographic and personal characteristics and anthropometric parameters of the study sample. The data are presented as numbers (N) and percentages (%) for categorical variables and as mean (M), standard deviation (SD), median (Mdn), and range (min-max) for continuous variables. For evaluating the difference in the levels of continuous variables across groups, the Mann-Whitney U test and the Kruskal-Wallis test were used as applicable. The Glass rank-biserial correlation r was reported for estimating the effect size for the Mann-Whitney U test and the eta squared (η2) was reported for the Kruskal-Wallis test [29]. The Spearman-Rho correlation coefficient was used to test the correlations between the continuous scale variables (scales of eating patterns, emotions, and loneliness). Multivariate regression analyses were subsequently conducted to investigate the ability of emotions and loneliness to predict eating patterns. Mediation analysis was also employed to assess whether loneliness has a mediating role in the relationship between emotions and eating patterns. The results were considered significant at p-values < 0.05. The statistical analysis was performed using SPSS ver. 25 (IBM Corp., Armonk, NY).

## Results

Demographic characteristics of the population

Table 1 presents the main demographic and socioeconomic characteristics of the study population. A predominance of women (77.1%) and of those aged under 30 years (48%) was observed, while approximately half of the responders were university graduates (53.6%). A total of 75.3% of the participants lived in an urban area, with one-third (29.3%) of the participants being married and 78.7% living with someone. A total of 45.6% of the sample had a monthly income of less than 1000 euros. 

**Table 1:** Demographic characteristics of the sample

Parameters	N	%
Gender		
Male	103	22.9
Female	347	77.1
Age		
18-24	57	12.7
25-29	160	35.6
30-34	67	14.9
35-40	62	13.8
40-44	42	9.3
45-49	23	5.1
50-54	14	3.1
55-59	14	3.1
60-64	7	1.6
65-69	3	0.7
70-74	1	0.2
Educational Status		
High School	57	12.7
Secondary School	5	1.1
University graduate	241	53.6
PhD graduate	6	1.3
Master's graduate	141	31.3
Place of Residence		
Rural	23	5.1
Urban	339	75.3
Semi-urban	88	19.6
Family Status		
Unmarried	299	66.4
Divorced	18	4.0
Married	132	29.3
Widowed	1	0.2
Number of children in family		
0	340	75.6
1	37	8.2
2	55	12.2
3	16	3.6
4	2	0.4
Living Conditions		
Living alone	96	21.3
Not living alone (with family/ friend/partner etc.)	354	78.7
Employment Status		
Unemployed	45	10.0
Employee	335	74.4
Household	6	1.3
Retired	9	2.0
College student	55	12.2
Household income per month (in euros)		
<500	71	15.8
500-999	134	29.8
1000-1499	100	22.2
1500-1999	60	13.3
2000-2499	37	8.2
2500-2999	14	3.1
3000+	34	7.6

Overall Levels of Positive/Negative Emotions and Loneliness

Regarding participants emotions, the mean (min-max) level was 34.9 (13.0-55.0) for the Positive Emotions scale (total possible lower and higher scores of the scale: 11-55) and 23.3 (9.0-43.0) for the Negative Emotions scale (total possible lower and higher scores of the scale: 9-45). Regarding Loneliness, the mean (min-max) score was 40.1 (20.0-79.0) (total possible lower and higher scores on the scale: 20-80).

Prevalence of Eating Patterns

One of the main goals of the study was to investigate whether eating patterns changed during quarantine. Table 2 presents the percentages of participants who adopted normal or abnormal eating patterns. We should highlight that 25% of the sample population had abnormal eating patterns.

**Table 2:** Prevalence of eating patterns in the sample EAT-26: Eating Attitudes Test

EAT-26	N	%
Normal eating patterns	336	74.7
Abnormal eating patterns	114	25.3

Research hypothesis 1

We initially investigated whether gender, age, and income differentiated individuals' eating patterns. According to Table 3 (Mann-Whitney U test), women had a greater score than men on the overall EAT-26 scale [Median (min-max)=15 (1.0-46.0) vs. 11 (2.0-32.0), U=14878.5, r=0.167, p=0.010) and on the Diet subscale [Median (min-max)=9.0 (0.0-33.0) vs. 8.0 (0.0-25.0), U=15341.5, r=0.142, p=0.029], although the strength of the relationship was of small magnitude.

**Table 3:** Evaluation of differences according to gender based on the Mann-Whitney U Test EAT-26: Eating Attitudes Test

Parameters	Gender	N	Mean (SD)	Median (Min-Max)	p-value
EAT-26 - Total	Male	103	13.8 (7.024)	11 (2.0-32.0)	0.010
	Female	347	16.2 (8.616)	15 (1.0-46.0)	
	Total	450	15.6 (8.332)	14.0 (1.0-46.0)	
Diet	Male	103	8.8 (5.878)	8.0 (0.0-25.0)	0.029
	Female	347	10.4 (6.588)	9.0 (0.0-33.0)	
	Total	450	10.1 (6.463)	9.0 (0.0-33.0)	
Bulimia	Male	103	1.8 (1.844)	1.0 (0.0-9.0)	0.323
	Female	347	2.4 (2.842)	1.0 (0.0-16.0)	
	Total	450	2.3 (2.658)	1.0 (0.0-16.0)	
Oral Control	Male	103	3.2 (2.864)	3.0 (0.0-18.0)	0.969
	Female	347	3.3 (3.156)	2.0 (0.0-16.0)	
	Total	450	3.3 (3.09)	2.0 (0.0-18.0)	
Positive Emotions	Male	103	33.4 (8.387)	33.0 (13.0-52.0)	0.086
	Female	347	35.3 (8.671)	35.0 (13.0-55.0)	
	Total	450	34.9 (8.634)	34.0 (13.0-55.0)	
Negative Emotions	Male	103	22.9 (7.093)	23.0 (9.0-39.0)	0.753
	Female	347	23.4 (6.958)	23.0 (10.0-43.0)	
	Total	450	23.3 (6.984)	23.0 (9.0-43.0)	
Loneliness	Male	103	41.0 (11.155)	41.0 (20.0-72.0)	0.221
	Female	347	39.8 (11.905)	38.0 (20.0-79.0)	
	Total	450	40.1 (11.736)	39.0 (20.0-79.0)	

Table 4 (Kruskal-Wallis test), displays the results pertaining to the reported changes in eating patterns according to age. A statistically significant difference of small magnitude was observed in the Diet subscale, with older participants having a higher score [Median (min-max)=8.0 (0.0-33.0) for 18-29 years, 9.0 (0.0-24.0) for 30-40 years and 10.0 (2.0-31.0) for over-40 years, chi-square=15.201, p=0.001, η2=0.03).

**Table 4:** Evaluation of differences according to age based on the Kruskal-Wallis test EAT-26: Eating Attitudes Test

Parameters	Age	N	Mean (SD)	Median (Min-Max)	p-value
EAT-26 - Total	18-29	217	15.4 (8.715)	15.0 (2.0-46.0)	0.075
	30-40	129	14.7 (7.013)	13.0 (1.0-36.0)	
	40+	104	17.4 (8.821)	14.5 (4.0-44.0)	
Diet	18-29	217	9.4 (6.587)	8.0 (0.0-33.0)	0.001
	30-40	129	9.4 (5.477)	9.0 (0.0-24.0)	
	40+	104	12.3 (6.874)	10.0 (2.0-31.0)	
Bulimia	18-29	217	2.4 (2.835)	1.0 (0.0-16.0)	0.554
	30-40	129	2.3 (2.405)	2.0 (0.0-12.0)	
	40+	104	2.1 (2.588)	1.0 (0.0-14.0)	
Oral Control	18-29	217	3.7 (3.334)	3.0 (0.0-18.0)	0.102
	30-40	129	2.9 (2.712)	2.0 (0.0-12.0)	
	40+	104	3.0 (2.94)	2.0 (0.0-12.0)	
Positive Emotions	18-29	217	35.1 (8.006)	35.0 (14.0-55.0)	0.558
	30-40	129	34.2 (8.809)	33.0 (13.0-54.0)	
	40+	104	35.2 (9.654)	35.0 (13.0-52.0)	
Negative Emotions	18-29	217	23.6 (6.225)	23.0 (9.0-41.0)	0.403
	30-40	129	22.9 (7.979)	22.0 (9.0-43.0)	
	40+	104	22.9 (7.195)	23.0 (10.0-42.0)	
Loneliness	18-29	217	39.6 (11.477)	38.0 (21.0-69.0)	0.485
	30-40	129	41.4 (12.797)	40.0 (20.0-79.0)	
	40+	104	39.6 (10.848)	40.0 (20.0-72.0)	

Table 5 (Kruskal-Wallis test) shows whether income played a role in shaping eating patterns. No differences were observed in the dietary attitudes of individuals according to income.

**Table 5:** Evaluation of differences according to income based on the Kruskal-Wallis test EAT-26: Eating Attitudes Test

Parameters	Income (in euros)	N	Mean (SD)	Median (Min-Max)	p-value
EAT-26 - Total	<500	71	15.4 (9.154)	14.0 (3.0-46.0)	0.237
	500-999	134	14.7 (8.051)	13.0 (1.0-41.0)	
	1000-1499	100	16.6 (7.734)	15.5 (2.0-38.0)	
	1500-1999	60	16.3 (7.784)	14.0 (3.0-34.0)	
	2000+	85	15.8 (9.094)	13.0 (4.0-44.0)	
Diet	<500	71	10 (6.994)	9.0 (0.0-33.0)	0.642
	500-999	134	9.4 (5.968)	8.0 (0.0-27.0)	
	1000-1499	100	10.6 (6.543)	9.0 (0.0-26.0)	
	1500-1999	60	10.3 (5.993)	10.0 (1.0-24.0)	
	2000+	85	10.4 (7.011)	8.0 (0.0-31.0)	
Bulimia	<500	71	2.4 (2.503)	2.0 (0.0-9.0)	0.759
	500-999	134	2.3 (2.808)	1.0 (0.0-16.0)	
	1000-1499	100	2.2 (2.629)	1.0 (0.0-12.0)	
	1500-1999	60	2.5 (2.432)	2.0 (0.0-9.0)	
	2000+	85	2.3 (2.776)	1.0 (0.0-14.0)	
Oral Control	<500	71	3.1 (2.756)	2.0 (0.0-15.0)	0.422
	500-999	134	3.0 (2.443)	3.0 (0.0-12.0)	
	1000-1499	100	3.8 (3.363)	4.0 (0.0-16.0)	
	1500-1999	60	3.5 (3.816)	2.0 (0.0-18.0)	
	2000+	85	3.1 (3.339)	2.0 (0.0-13.0)	
Positive Emotions	<500	71	32 (8.225)	31.0 (14.0-55.0)	0.01
	500-999	134	34.4 (8.103)	33.0 (14.0-54.0)	
	1000-1499	100	36.6 (8.436)	38.0 (18.0-52.0)	
	1500-1999	60	35.4 (8.914)	34.5 (20.0-53.0)	
	2000+	85	35.7 (9.32)	35.0 (13.0-54.0)	
Negative Emotions	<500	71	25.8 (7.576)	26.0 (11.0-41.0)	0.019
	500-999	134	23.3 (6.205)	23.0 (12.0-41.0)	
	1000-1499	100	23.0 (7.165)	23.0 (10.0-43.0)	
	1500-1999	60	21.7 (6.668)	21.0 (11.0-38.0)	
	2000+	85	22.5 (7.24)	23.0 (9.0-41.0)	
Loneliness	<500	71	45.1 (10.582)	43.0 (20.0-68.0)	<0.001
	500-999	134	41.0 (13.031)	40.0 (21.0-78.0)	
	1000-1499	100	37.1 (9.096)	36.0 (20.0-72.0)	
	1500-1999	60	39.5 (12.950)	35.5 (20.0-68.0)	
	2000+	85	38.5 (11.078)	38.0 (23.0-79.0)	

Research hypothesis 2

We also investigated whether emotions and loneliness were predictive factors of eating attitudes. First, Table 6 shows that correlations between eating patterns and emotions and loneliness actually existed. There were some statistically significant correlations between eating attitudes (Total Score, Diet, and Bulimia subscales) and the scores for the Negative and Positive Emotions subscale and Loneliness scale, but these correlations were extremely close to 0.

**Table 6:** Correlations (Spearman’s rho) between the EAT-26, Emotions, and Loneliness scales *  Level of significance 10% ** Level of significance 5% EAT-26: Eating Attitudes Test

Parameters		Positive Emotions	Negative Emotions	Loneliness
EAT-26 - Total	rho	-0.123^**^	0.165^**^	0.164^**^
	p-value	0.009	<0.001	<0.001
Diet	rho	-0.131^**^	0.127^**^	0.106^*^
	p-value	0.005	0.007	0.024
Bulimia	rho	-0.038	0.194^**^	0.189^**^
	p-value	0.423	<0.001	<0.001
Oral Control	rho	0.005	-0.002	0.042
	p-value	0.916	0.974	0.372

In addition, linear regression analysis was performed. Table 7 indicates whether positive and negative emotions and loneliness were predictive factors of eating attitudes not only in general but also, especially, in each separate subscale of the EAT-26. The Negative Emotions (β=0.128, p=0.035) and Loneliness (β=0.099, p=0.011) subscales were found to be predictors of the overall EAT-26 score (R^2^=4.8%). The Negative Emotions (β= 0.047, p=0.015) and Loneliness (β=0.038, p=0.002) subscale scores were predictors of the Bulimia subscale score (R^2^=5.3%); however, this finding should be interpreted with caution since the assumptions of the normality of residuals and homoscedasticity of the residuals were not sufficiently met for this subscale.

**Table 7:** Multivariate regression analysis: predicting eating patterns (EAT-26 Overall score and Diet, Bulimia, Oral Control subscales) from emotions and loneliness *R^2^=4.8%, **R^2^= 2.8%, ***R^2= ^5.3%, **** R^2^= 0.8% EAT-26: Eating Attitudes Test

Parameters	β	CI 95%	p-value
Independent Variable EAT-26 (Total)			
Positive Emotions	-0.025	-0.122, 0.071	0.604
Negative Emotions	0.128	0.009, 0.247	0.035
Loneliness	0.099	0.023, 0.175	0.011*
EAT- 26 (Diet)			
Positive Emotions	-0.055	-0.131, 0.021	0.153
Negative Emotions	0.082	-0.011, 0.175	0.083
Loneliness	0.035	-0.025, 0.094	0.255**
EAT-26 (Bulimia)			
Positive Emotions	0.017	-0.013, 0.048	0.27
Negative Emotions	0.047	0.009, 0.085	0.015
Loneliness	0.038	0.014, 0.063	0.002***
EAT-26 (Oral Control)			
Positive Emotions	0.012	-0.024, 0.049	0.508
Negative Emotions	-0.001	-0.046, 0.044	0.972
Loneliness	0.026	-0.003, 0.055	0.078****

Research hypothesis 3

Finally, we examined whether loneliness mediated the relationship between positive/negative emotions and eating patterns. The overall EAT-26 score and the score on the Bulimia subscale were examined because statistically significant correlations were observed during the linear regression analysis, as presented in Table 7. Descriptive statistics of the Loneliness scale according to the demographic factors are presented in Table 8.

**Table 8:** Descriptive statistics of the Loneliness scale according to demographic factors

Parameters	N	Loneliness Scale	
		Mean (SD)	Median (Min-Max)
Gender			
Male	103	41.0 (11.155)	41.0 (20.0-72.0)
Female	347	39.8 (11.905)	38.0 (20.0-79.0)
Age group			
18-29	217	39.6 (11.477)	38.0 (21.0-69.0)
30-40	129	41.4 (12.797)	40.0 (20.0-79.0)
40+	104	39.6 (10.848)	40.0 (20.0-72.0)
Education Status			
Secondary School	5	41.4 (5.177)	43.0 (35.0-47.0)
High School	57	43.1 (13.078)	43.0 (23.0-79.0)
University graduate	241	40.6 (11.847)	40.0 (20.0-78.0)
Master's graduate	141	38.1 (10.910)	35.0 (21.0-71.0)
PhD graduate	6	39.8 (11.409)	39.5 (26.0-60.0)
Place of Residence			
Rural area	23	38.2 (10.594)	36.0 (22.0-69.0)
Urban area	339	40.1 (11.644)	40.0 (20.0-78.0)
Semi-urban area	88	40.5 (12.435)	38.5 (20.0-79.0)
Family Status			
Unmarried	299	40.9 (11.798)	40.0 (21.0-78.0)
Divorced	18	43.8 (13.085)	43.0 (20.0-72.0)
Married	132	37.8 (11.153)	35.5 (20.0-79.0)
Widower	1	-	-
Number of children in family			
0	340	40.6 (11.753)	40.0 (21.0-78.0)
1	37	38.0 (10.349)	34.0 (23.0-68.0)
2	55	40.1 (13.007)	40.0 (20.0-79.0)
3	16	34.1 (8.709)	32.0 (20.0-52.0)
4	2	39.0 (8.485)	39.0 (33.0-45.0)
Living Conditions			
Living alone	96	41.8 (11.914)	41.0 (23.0-72.0)
Not living alone (with family/ friend/ partner etc.)	354	39.6 (11.664)	39.0 (20.0-79.0)
Employment status			
Unemployed	45	46.6 (12.095)	46.0 (23.0-68.0)
Employee	335	39.6 (11.634)	39.0 (20.0-79.0)
Household	6	41.7 (13.246)	38.0 (31.0-67.0)
Retired	9	35.4 (11.215)	33.0 (23.0-56.0)
College student	55	38.3 (10.500)	36.0 (22.0-69.0)
Household income per month			
<500	71	45.1 (10.582)	43.0 (20.0-68.0)
500-999	134	41.0 (13.031)	40.0 (21.0-78.0)
1000-1499	100	37.1 (9.096)	36.0 (20.0-72.0)
1500-1999	60	39.5 (12.950)	35.5 (20.0-68.0)
2000+	85	38.5 (11.078)	38.0 (23.0-79.0)

There was no direct effect of positive emotions on the overall EAT-26 score (direct effect p-value=0.531; Table 9), which is in line with the findings of linear regression (Table 7). Regarding the relationship between negative emotions and the overall EAT-26 score (Table 9), negative emotions seemed to have a direct effect on the EAT-26 score (direct effect p-value=0.032); however, there was also significant mediating effect of loneliness. Therefore, the relationship between negative emotions and the overall EAT-26 score was partly mediated by loneliness.

**Table 9:** Results of mediation analysis (Y=ΕΑΤ-26 Bulimia, M=Loneliness)

	β	SE (β)	p-value
Χ=Positive Emotions			
Total effect on Y	-0.0125	0.0145	0.391
Direct effect on Y	0.0153	0.0157	0.531
Indirect effect on Y	-0.0278	0.0071	<0.001
Χ=Negative Emotions			
Total effect on Y	0.0686	0.0177	<0.001
Direct effect on Y	0.0456	0.0192	0.032
Indirect effect on Y	0.0229	0.0082	0.005

Table 10 shows that there was no direct effect of positive emotions on the Bulimia subscale score (direct effect p-value=0.330), which is in line with the linear regression findings presented in Table 7. Regarding the relationship between the Negative Emotions and the Bulimia subscale scores (Table 10), negative emotions seemed to have a direct effect on bulimia (direct effect p-value=0.018); however, there was also a significant mediating effect of loneliness. Thus, the relationship between Negative Emotions and the Bulimia subscale score is mediated in part by loneliness.

**Table 10:** Results of mediation analysis (Y=ΕΑΤ-26 total score, M= Loneliness).

	β	SE (β)	p-value
Χ= Positive Emotions			
Total effect on Y	-0.1032	0.0453	0.023
Direct effect on Y	-0.0309	0.0492	0.330
Indirect effect on Y	-0.0724	0.0219	<0.001
Χ= Negative Emotions			
Total effect on Y	0.2026	0.0555	<0.001
Direct effect on Y	0.1297	0.0603	0.018
Indirect effect on Y	0.0728	0.0259	0.005

## Discussion

Quarantine can be classified as a stressful event, and such events can affect the eating patterns of individuals [10]. The present sample showed that most participants had normal eating patterns. However, 25% of the participants had abnormal eating patterns, while a cohort study in the UK revealed a greater rate of eating changes [30]. The participants tended to adopt more normal dietary behaviors rather than bulimic behaviors. Sex is considered an important differentiating factor that affects human behavior [3, 8, 9]. However, a review of the present literature indicated that the female sex is more often associated not only with the occurrence of an eating disorder but also with self-limiting food behaviors in general and episodes of overeating [14]. Female sex is more strongly associated with loneliness [7] and is generally associated with lower levels of mental health [8, 9]. This finding was indeed confirmed by the present study, which showed that women had more abnormal eating patterns than men did and, in fact, had more frequent dietary behaviors. Notably, in the present sample, participants over 40 years old exhibited more dietary behaviors than younger participants. Low income and loss of income are also factors that can affect the onset of severe anxiety and depression, while unemployment and low household income are risk factors for higher levels of loneliness [7, 19]. Income, however, did not appear to play a role in shaping eating patterns in the present study.

Emotions play a leading role in the way we perceive and interpret the world around us, and eating behaviors and emotional factors seem to be clearly linked. Food intake can increase or decrease when people experience stressful or negative situations, depending on external or psychological stressors [10, 31]. The present study showed that negative emotions are a predictor of eating patterns in general and bulimic behaviors in particular. This finding is in line with previous research showing that people with episodic overeating expressed a greater desire for food when they experienced negative emotions [32].

Furthermore, this study attempted to investigate whether loneliness is a predictor of eating patterns. Indeed, loneliness, as well as negative emotions in general, seemed to be a predictor of eating patterns, and it was observed that loneliness better predicts involvement in bulimic behaviors. Increased social isolation and loneliness need to be included among the most significant effects of the COVID-19 pandemic [17]. Food seems to have an emotional quality that leads to "numbing" emotions, including loneliness [20]. Negative emotions seem to be associated with overeating [21, 32]. Finally, for the first time, our study attempted to investigate whether loneliness mediates the relationship between positive or negative emotions and eating patterns. While negative emotions directly affected the formation of eating patterns, feelings of loneliness had a significant mediating effect. Therefore, our definitive conclusion is that loneliness can affect a person’s eating patterns, especially when negative emotions coexist. In conjunction with a sense of loneliness, negative emotions significantly affect people’s eating behaviors. In fact, loneliness was also a mediating variable in the relationship between negative emotions and bulimia. Negative emotions are therefore more likely to lead to bulimic behaviors; however, a person's sense of loneliness also seems to regulate the intensity of this relationship.

Strengths and limitations

Despite the insightful results of our study, there are several limitations. First, the sample was imbalanced in terms of sex and age. In addition, the selection of the sample did not result from random sampling, so the generalizability of the results to the whole population is limited. Furthermore, the study was based on data from self-report questionnaires, in which individuals may give false information or information that is socially acceptable, which negatively affects the validity of the results. Additionally, the regression analysis results regarding the association of EAT-26 Bulimia and Oral Control scales should be interpreted with caution, since the assumptions of normality and homoscedasticity of the residuals were not sufficiently met. Finally, we used a cross-sectional design, so we cannot evaluate causal relationships between the studied indicators. This study is one of the very few studies that has focused on eating patterns during quarantine in Greece, exploring the role of emotions and loneliness, which are research areas that need more thorough research. Furthermore, the conduction of mediational analysis could be considered a significant contribution; firstly, no similar analysis was available previously, and secondly, highlights the pivotal role of loneliness in the relationship between emotions and eating patterns.

## Conclusions

During the spread of major infectious diseases, quarantine can be a necessary preventive measure. This research, as well as a review of previous research, reveals that quarantine and social exclusion are often associated with negative psychological effects. Eating attitudes seem to be strongly affected during stressful periods, as food has psychological qualities for many people. Negative emotions directly affect the formation of eating patterns, especially bulimic behaviors. In particular, our study findings show that loneliness predicts overeating attitudes better than dieting and seems to mediate the connection between negative emotions and eating patterns. Clinical experience has shown that, like many others, eating disorders are the tip of the iceberg. The challenge for public health officials is to promote coping strategies that can assist individuals in determining the source of their personal discomfort.
